# A Subset of CXCR5^+^CD8^+^ T Cells in the Germinal Centers From Human Tonsils and Lymph Nodes Help B Cells Produce Immunoglobulins

**DOI:** 10.3389/fimmu.2018.02287

**Published:** 2018-10-05

**Authors:** Juan Shen, Xi Luo, Qiongli Wu, Jun Huang, Guanying Xiao, Liantang Wang, Binyan Yang, Huabin Li, Changyou Wu

**Affiliations:** ^1^Guangdong Provincial Key Laboratory of Organ Donation and Transplant Immunology, Zhongshan School of Medicine, Institute of Immunology, Sun Yat-sen University, Guangzhou, China; ^2^Affiliated Guangzhou Women and Children's Medical Center, Guangzhou Medical University, Guangzhou, China; ^3^Department of Pathogenic Biology and Immunology, Institute of Immunology, Guangzhou Medical University, Guangzhou, China; ^4^Guangzhou Eighth People's Hospital, Guangzhou Medical University, Guangzhou, China; ^5^Department of Pathology, The First Affiliated Hospital, Sun Yat-sen University, Guangzhou, China; ^6^Eye and Ent Hospital of Fudan Hospital, Shanghai, China

**Keywords:** CD8 T cell, C-X-C chemokine receptor type 5, Follicular, B cell, Tfh-like cell

## Abstract

Recent studies indicated that CXCR5^+^CD8^+^ T cells in lymph nodes could eradicate virus-infected target cells. However, in the current study we found that a subset of CXCR5^+^CD8^+^ T cells in the germinal centers from human tonsils or lymph nodes are predominately memory cells that express CD45RO and CD27. The involvement of CXCR5^+^CD8^+^ T cells in humoral immune responses is suggested by their localization in B cell follicles and by the concomitant expression of costimulatory molecules, including CD40L and ICOS after activation. In addition, CXCR5^+^CD8^+^ memory T cells produced significantly higher levels of IL-21, IFN-γ, and IL-4 at mRNA and protein levels compared to CXCR5^−^CD8^+^ memory T cells, but IL-21-expressing CXCR5^+^CD8^+^ T cells did not express Granzyme B and perforin. When cocultured with sorted B cells, sorted CXCR5^+^CD8^+^ T cells promoted the production of antibodies compared to sorted CXCR5^−^CD8^+^ T cells. However, fixed CD8^+^ T cells failed to help B cells and the neutralyzing antibodies against IL-21 or CD40L inhibited the promoting effects of sorted CXCR5^+^CD8^+^ T cells on B cells for the production of antibodies. Finally, we found that in the germinal centers of lymph nodes from HIV-infected patients contained more CXCR5^+^CD8^+^ T cells compared to normal lymph nodes. Due to their versatile functional capacities, CXCR5^+^CD8^+^ T cells are promising candidate cells for immune therapies, particularly when CD4^+^ T cell help are limited.

## Introduction

CD8^+^ T cells constitute an important branch of adaptive immunity contributing to recognizing viral and bacterial peptides presented by MHC class I molecules on the target cells, and to eliminating of intracellular pathogens and tumors (1–5). Those biological functions of CD8^+^ T cells are mostly fulfilled by their ability to kill infected cells via the targeted release of the lytic molecules, perforin and granzymes (6–8), and to secrete cytokines such as IFN-γ and TNF-α (9–11). In addition, two studies (12, 13) demonstrated that CD8^+^ T cells from human tonsils help B cells produce immunoglobulins, suggesting a significant heterogeneity in the differentiation and function of CD8^+^ T cells.

CXCR5 is a chemokine receptor expressed on B cells, as well as dendritic cells (DCs) and T cell subsets (12, 14, 15). The chemokine CXCL13 mainly produced by stromal cells and follicular DCs in the germinal center is the ligand to CXCR5. CXCR5^+^ CD4^+^ follicular helper T (Tfh) cells are considered the main helper cells to B cells and are critical for activation of B cells, antibody class switching and germinal center formation (14–18). In recent years, a subset of CD8^+^ T cells with high expression of CXCR5 is observed in human secondary lymphoid tissues such as lymph nodes, tonsils and spleens (19–25). Those CXCR5^+^CD8^+^ T cells possess proinflammatory functions with theraputic potential in multiple inflammatory diseases. In a murine LCMV model, He et al, suggested that compared to CXCR5^−^CD8^+^ T cells, the CXCR5^+^CD8^+^ T cells expressed lower level of T cell exhaustion markers, PD-1 and Tim-3, and higher level of proinflammatory cytokines IFN-γ and TNF-α (24). The CXCR5^+^CD8^+^ T cells presented cytotoxic potential toward virus-infected Tfh cells and B cells, and could be localized in proximity with HIV-infected cells in the lymph nodes from an HIV-infected human subjects (25).

In the current study, we found that a subset of human CD8^+^ T cells in tonsils and lymph nodes expressed CXCR5 to localize in B cell follicles. CXCR5^+^CD8^+^ T cells expressed co-stimulatory molecules including ICOS and CD40L, which interact with their corresponding ligands on B cells, and secreted IL-21, which could help B cells in the germinal center for antibody production. Together, our results indicated that CXCR5^+^CD8^+^ T cells more like CXCR5^+^CD4^+^ T cells in the germinal centers could help B cells to produce antibodies.

## Materials and methods

### Study participants

Tonsil tissues from 67 individuals with tonsillar hypertrophy were obtained from patients undergoing tonsillectomy at Guangzhou Women and Children's Medical Center of Guangzhou Medical University. Samples of normal lymph nodes were obtained from 10 patients with colorectal cancer undergoing curative resection at the sixth Affiliated Hospital of Sun Yat-sen University. Paraffin sections of lymph nodes from HIV patients were obtained from Guangzhou Eighth People's Hospital. Twenty healthy volunteers were recruited from Blood Center of Guangzhou. Written informed consent was obtained from all individuals, and the protocol was approved by the Review Board of Sun Yat-sen University.

### Isolation of mononuclear cells from peripheral blood and tissues

Tonsils and lymph nodes were processed immediately by mashing through a 40-μm nylon filter (BD Bioscience, San Diego, CA, USA) with Hank's balanced salt solution. The resulting cell suspension was isolated by ficoll-hypague (Tianjin Hao Yang Biological Manufacture, Tianjin, China) density gradient centrifugation at 400 g for 20 min. The mononuclear cells were collected and washed twice. PBMCs was similarly isolated over a lymphoprep gradient. The cells were suspended at a final concentration of 2 × 10^6^ cells/mL in completed RPMI-1640 medium (Gibco, Grand Island, USA) supplemented with 10% heat-inactivated fetal calf serum (Sijiqing, Hangzhou, China), 100 μg/mL streptomycin, 100 U/mL penicillin, 2 mM L-glutamine and 50 μM 2-mercaptoethanol (all from Gibco).

Purification of B cells, CD4^+^ and CD8^+^ T cells from tonsils was achieved with a MACS column purification system (Miltenyi Biotec, Auburn, USA). B cells, CD4^+^ cells, CD8^+^ cells, CXCR5^+^CD8^+^ T cells, and CXCR5^−^CD8^+^T cells were further sorted by using FACS Arial II (BD Bioscience, San Deigo, CA, USA). B cells of >98% purity and >90% viability, CD8^+^T cells of >98% purity and >90% viability, CXCR5^+^CD8^+^T cells of >97% purity and >90% viability, and CXCR5^−^CD8^+^T cells of >98% purity and >90% viabillity were used for subsequent functional experiments.

### Flow cytometry and MAbs

The procedures for studying cell phenotype, intracellular cytokines, and transcriptional factor expression had been previously described (26). Generally, cells were washed and suspended in 100 μl of PBS containing 0.1% BSA and 0.05% sodium azide. For surface staining, cells were incubated with the respective mAbs at 4°C in the dark for 30 min. For the detection of intracellular cytokines, cells were fixed with 4% paraformaldehyde and permeabilized in PBS buffer containing 0.1% saponin (Sigma-Aldrich, St Louis, MO, USA), 0.1% BSA and 0.05% NaN_3_ for at least 2 h or overnight at 4°C and stained with conjugated mAbs for intracellular cytokines. For the detection of intracellular transcription factors, cells were stained for surface antigens, followed by fixation, permeabilization with Permeabilization/Fixation buffer (BD Bioscience) and staining according to the protocol of Permeabilization/Fixation Kit. Flow cytometry data were acquired with a FACS Arial II (BD Bioscience) and were analyzed with FlowJo software (Tree Star, San Carlos, CA, USA). The increase in mean fluorescence intensity (ΔMFI) was calculated as: (MFI (specific mAb)-MFI (isotype control))/MFI (isotype control).

The following mAbs were used for cell surface or intracellular stainings: Phycoerythrin-Texas Red (ECD)-conjugated anti-CD3, Alexa Fluor 700 (AF700)-conjugated anti-CD8, anti-CD45RO, Phycoerythrin (PE)-conjugated anti-CD27, anti-CD62L, anti-CCR7, anti-CD40L, anti-IL-4, anti-T-bet, anti-Granzyme B, Alexa Fluor 488 (AF488)-conjugated anti-CXCR5, Allophycocyanin (APC)-conjugated anti-ICOS, anti-Perforin, anti-IL-21, Phycoerythrin-cyanin (PE-Cy7)-conjugated anti-CD45RO, anti-IFN-γ, Fluorescein isothiocyanate (FITC)-conjugated anti-Bcl-6, and isotype-matched control antibodies were purchased from BD Bioscience (San Deigo, CA, USA).

### Cell culture

To analyze the expression of cytokines and transcription factors, the lymphocytes in tonsils, lymph nodes and PBMCs were stimulated for 5 h with PMA (20 ng/ml; Sigma-Aldrich) and ionomycin (1 μg/ml; Sigma-Aldrich) at 37°C with 5% CO2 in the presence of brefeldin A (BFA, 10 μg/ml; Sigma-Aldrich). Sorted CD8^+^, CXCR5^+^, or CXCR5^−^ CD8^+^ T cells were cultured for 48 h with PMA and ionomycin. Cell-free supernatants were harvested and assayed by ELISA for the production of IL-21, IFN-γ, and IL-4. For functional assays, sorted CD8^+^, CXCR5^+^, or CXCR5^−^CD8^+^ T cells were co-cultured with purified B cells in the presence of T-activator CD3/CD28 dynabeads (Gibco, Grand Island, USA) at a ratio of 1:5 B cells per T cells for 10 days, unless otherwise indicated. In some experiments, T cells were pre-incubated with a blocking Ab against IL-21 (1 μg/ml; Peprotech, Rocky Hill, USA) and CD40L (100 ng/ml; eBioscience).

### Immunofluorescence staining

Paraffin sections of tonsil tissues and lymph nodes from cancer patients and HIV patients were rehydrated and boiled in EDTA buffer (pH8.0) for 20 min to induce antigen retrieval. After washing, tissue sections were blocked with 5% goat serum, followed by staining with rabbit anti-human CD3 antibody (clone: EP41, ZSGB, Beijing, China) or rabbit anti-human CXCR5 antibody (clone: EPR8837, 1:200, abcam) and mouse anti-human CD8 antibody (clone: C8/144B, MXB Biotechnologies, Fuzhou, China) at 4°C overnight. Sections were washed and incubated with Alexa Fluor 488–conjugated anti-rabbit IgG plus Alexa Fluor 555–conjugated anti-mouse IgG, or Alexa Fluor 488–conjugated anti-rabbit IgG plus Alexa Fluor 555–conjugated anti-goat IgG (Beyotime, Shanghai, China) for 30 min at 37°C in the dark. After a final washing, cover slips were mounted onto slides with fluoroshield mounting medium with DAPI (4, 6-diamidino-2-phenylindole; abcam). Images were captured with Olympus microscope BX53 and processed with LSM Image Examiner software (Zeiss).

### ELISA and elispot assays

For the quantitative ELISA assay of cytokines, sorted CD8^+^, CXCR5^+^, or CXCR5^−^CD8^+^ T cells were cultured with or without PMA and ionomycin for 48 h. The culture supernatants were collected for the quantitative assessment of IL-21 with Human IL-21 ELISA Ready-SET-Go!® (eBioscience), IFN-γ and IL-4 with BD OptEIA^TM^ Human ELISA Sets (BD Bioscience) according to the manufacturer's instructions. For the ELISA assay of immunoglobulins, co-culture supernatants from B cells and T cells were harvested, and the levels of IgG, IgM, and IgA were measured by Human IgG, IgM or IgA ELISA Ready-SET-Go!® (eBioscience). For the ELISPOT assay of cytokine-producing cells, sorted CD8^+^, CXCR5^+^ or CXCR5^−^CD8^+^ T cells were suspended in complete RPMI-1640 medium at a density of 1 × 10^6^/ml and stimulated with PMA plus ionomycin in pre-coated plates,100 μl/well for 16 h. The frequency of IL-21 or IFN-γ-producing cells was counted using an ImmunoSpot S6 Analyzer (Cellular Technology Ltd., USA) according to the manufacturer's instructions, and the results were shown as the mean of readings from triplicate wells.

### RT-PCR

For comparison of gene expression, CD8^+^, CXCR5^+^ and CXCR5^−^CD8^+^ T cells were sorted and stimulated with or without PMA plus ionomycin for 1 h. Total RNA was extracted in Trizol LS reagent (Life Technologies) and reverse-transcribed with a PrimeScript ^TM^ RT reagent Kit (TaKaRa, Japan). Amplification of cDNA was conducted in a DNA thermal cycler (Biometra, Germany). The following sense and antisense primers for each molecule were used: IL-21 Forward: 5′-GAGTGGTCAGCTTTTTCCTGTT-3′, reverse:5′-AGGAATTCTTTGGGTGGTTTTT-3′; IFN-γ Forward: 5′-TGGCTTTTCAGCTCTGCATCGT-3′,reverse:5′-TCCACACTCTTTTGGATGCTCTGGT-3′; glyceraldehyde-3-phosphate dehydrogenase (GAPDH) forward: 5′-GCATGGCCTTCCGTGTCC-3′, reverse: 5′-TGAGTGTGGCAGGGACTC-3′. The ratio of IL-21 or IFN-γ over GAPDH was calculated according to the relative intensities of the bands revealed under UV illumination with Bio-1D software (Vilber Lourmat, Marne la Vallee, France).

### Statistical analysis

Data were presented as the mean ± standard deviation (S.D.). Statistical significance was analyzed by Mann-Whitney test and two-tailed unpaired *t*-test (95% confidence interval) using Prism 5 (GraphPad, San Diego, CA, USA). Significant *p*-values are indicated in figures for the following ranges: ^*^P < 0.05; ^**^P < 0.01; ^***^P < 0.001.

## Results

### CD8^+^ T cells expressed CXCR5 to localize in B cell follicles

**Figure 1:**
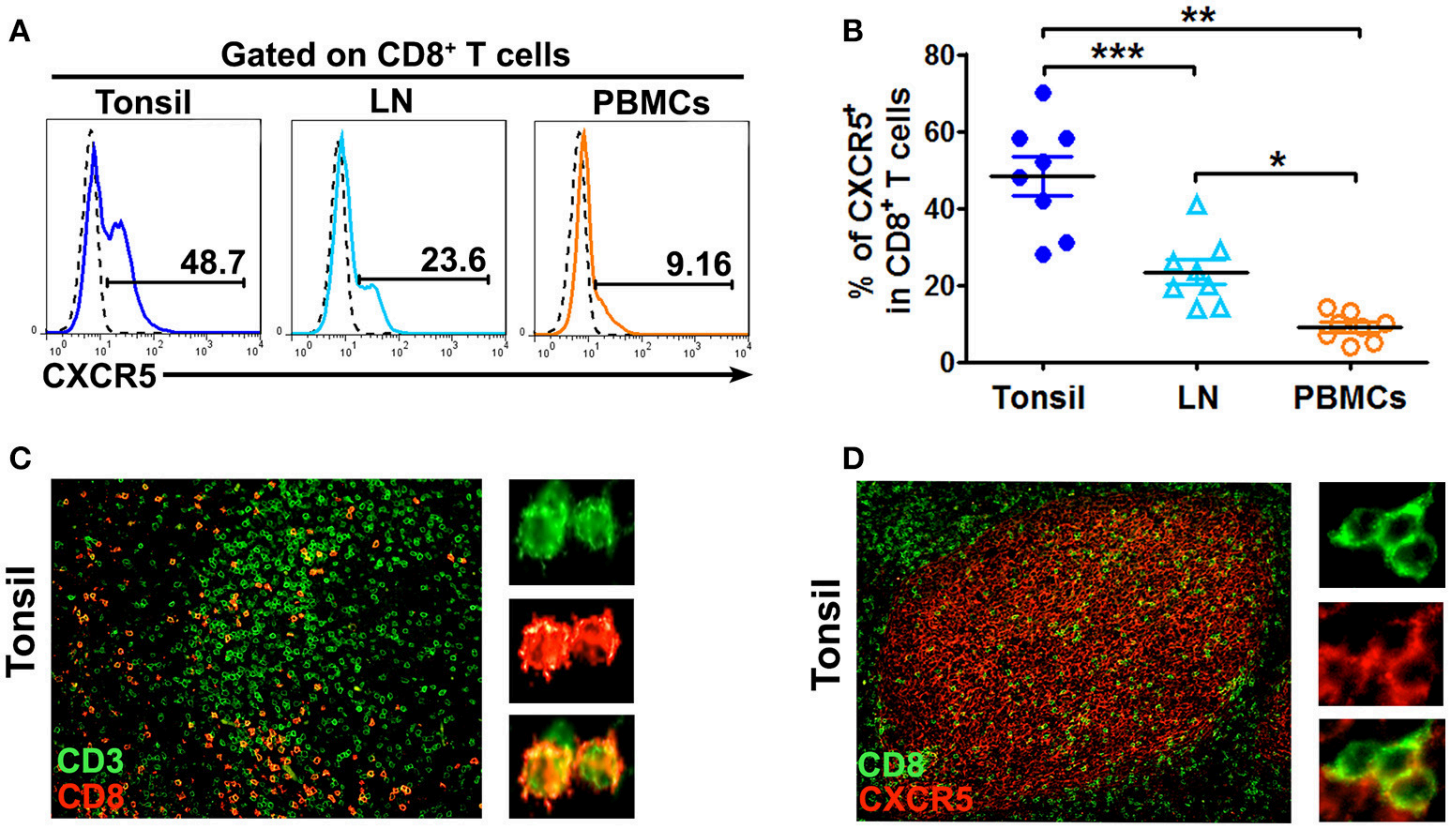
CD8^+^ T cells localized in B cell follicles in tonsils and lymph nodes express CXCR5. The expression of CXCR5 on CD8 T cells in tonsils, lymph nodes and PBMCs was shown in the representative histogram graphs **(A)** and summary data **(B**, *n* = 8). Immunofluorescence staining of CD3^+^ T cells (green) and CD8^+^ T cells (red) **(C**, *n* = 5), CD8^+^ T cells (green) and CXCR5 (red) **(D**, *n* = 5) in paraffin-embedded tonsil tissues. Data are expressed as the mean ± SD, and compared with Mann-Whitney test. *P < 0.05, **P < 0.01, and ***P < 0.001.

The mononuclear cells from human tonsils, lymph nodes and PBMCs were stained with anti-CD3, anti-CD8 and anti-CXCR5 mAbs and gated on CD8^+^ T cells. The results showed that 48.7% of CD8^+^ T cells from tonsils expressed CXCR5, which was significantly higher than those from lymph nodes (23.6%, *P* < 0.001) and PBMCs (9.16%, *P* < 0.01) (Figures 1A,B). To find out the distribution of CD8^+^ T cells in tonsil lymphoid tissues, immunofluorescence analysis of paraffin tonsil sections confirmed that CD8^+^ T cells were found dispersed in tonsil B cell follicles (Figure 1C) and co-expressed the chemokine receptor CXCR5 (Figure 1D).

To identify the memory phenotype of CXCR5^+^ or CXCR5^−^CD8^+^ T cells in tonsil tissues, we analyzed the expression of CD45RO, CCR7, CD62L, and CD27 by flow cytometry. Notably, CXCR5^+^ CD8^+^ T cells predominantly expressed CD45RO and CD27 but CXCR5^+^CD8^+^ T cells expressed lower levels of CCR7 and CD62L than did CXCR5^−^CD8^+^ T cells (Figures S1A-C). Taken together, these data suggested that most of CXCR5^+^CD8^+^ T cells in tonsils are effector or central memory cells.

### CXCR5^+^CD8^+^ memory T cells from tonsils and lymph nodes expressed costimulatory molecules

**Figure 2:**
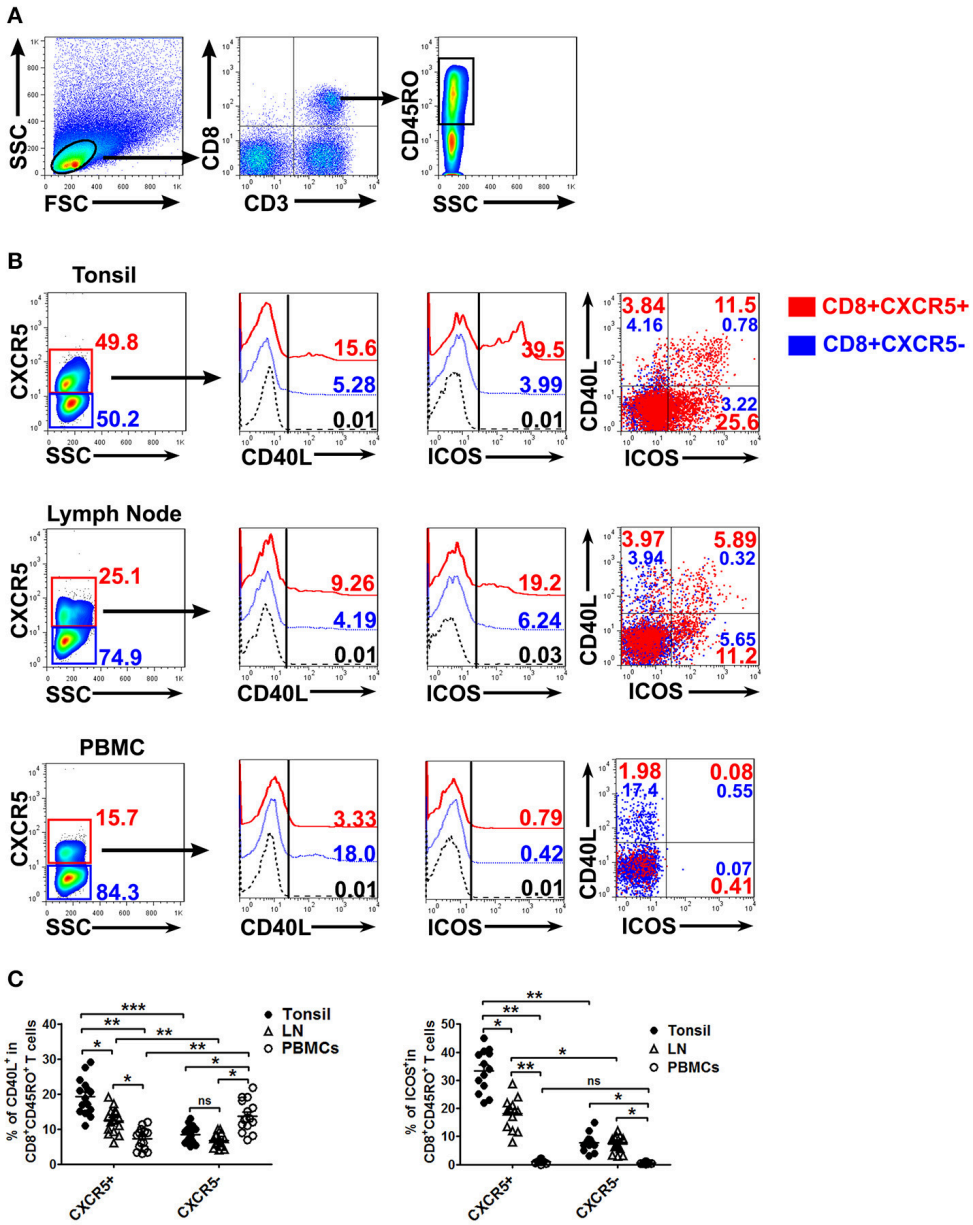
Expression of co-stimulated molecules on CXCR5^+^ CD8^+^ memory T cells from tonsils, lymph nodes and PBMCs. Upon stimulation with CD3/CD28 dynabeads for 8 h, the expression of CD40L and ICOS on CXCR5^+^ and CXCR5^−^ CD8^+^ memory T cells from tonsils, lymph nodes and PBMCs was detected by flow cytometry **(A,B)**. The data are representative of thirteen or fifteen independent experiments, and were analyzed by two-tailed unpaired *t*-test **(C)**. Error bar denote s.e.m. *P < 0.05, **P < 0.01, and ***P < 0.001. ns, no significance.

To investigate the expression of CD40L and ICOS, cells from tonsils, lymph nodes and PBMCs were stimulated with CD3/CD28 dynabeads for 8 h. The cells were then harvested, stained and analyzed by flow cytometry. The results showed that CXCR5^+^CD8^+^ memory T cells in tonsils and lymph nodes expressed significantly higher levels of CD40L and ICOS than did CXCR5^−^CD8^+^ memory T cells (*P* < 0.01), and also had higher levels of CD40L^+^ICOS^+^ (Figures 2A–C, *P* < 0.05). By contrast, the expression of CD40L on CXCR5^+^CD8^+^ memory T cells was clearly lower than that on CXCR5^−^CD8^+^ memory T cells in PBMCs, and the levels of ICOS expression in PBMCs had no statistical significance between CXCR5^+^ and CXCR5^−^CD8^+^ memory T cells (Figures 2A–C).

### CXCR5^+^CD8^+^ memory T cells from tonsils and lymph nodes expressed high levels of IL-21, IL-4, and IFN-γ

**Figure 3:**
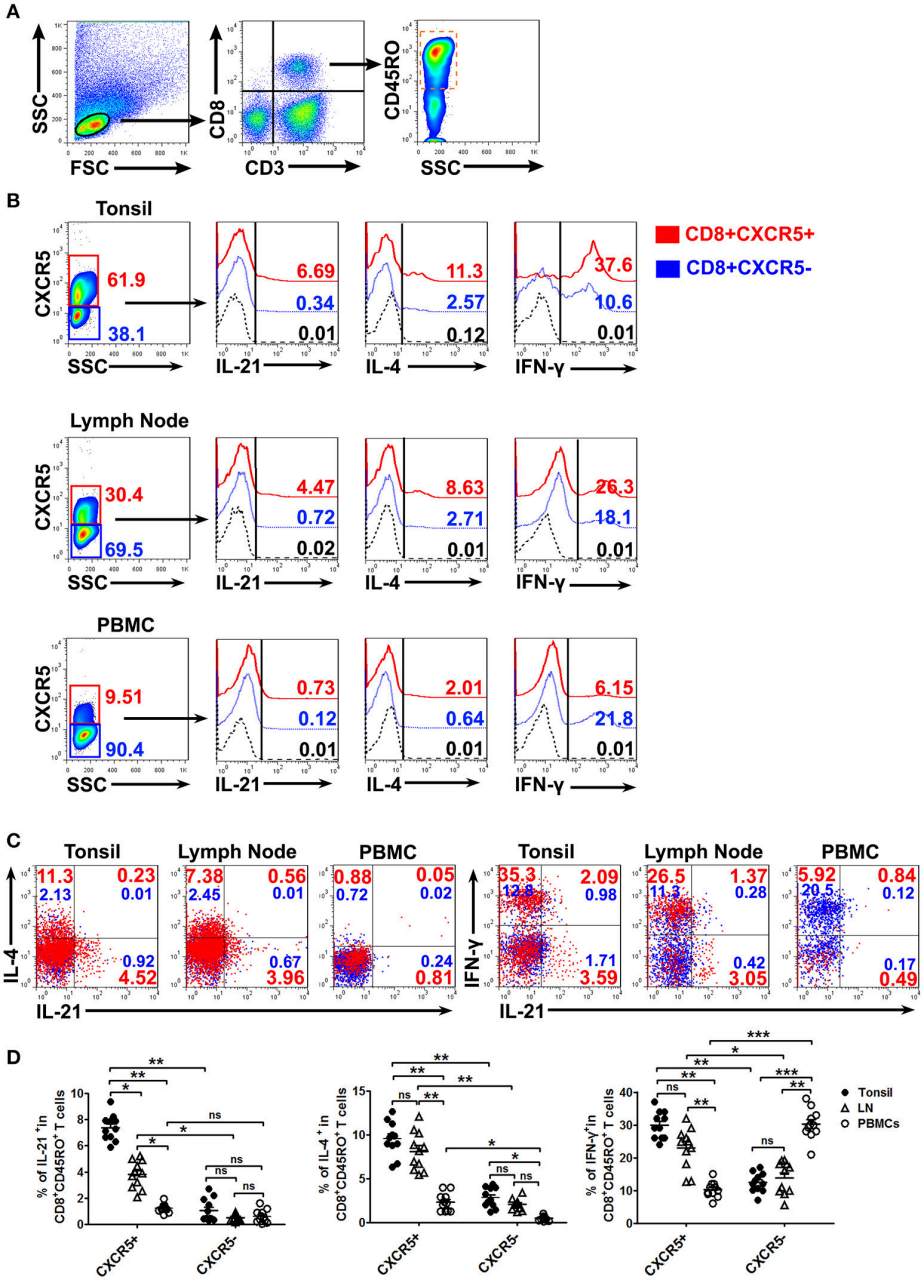
The expression of cytokines by CXCR5^+^ CD8^+^ memory T Cells from tonsils, lymph nodes and PBMCs. The mononuclear cells from tonsils, lymph nodes and blood were cultured with or without PMA plus ionomycin in the presence of BFA for 6 h. FACS analysis of IL-21, IL-4, and IFN-γ expression in CXCR5^+^ and CXCR5^−^ CD8^+^ memory T Cells **(A–C)**. Data represent mean ± SD, and compared with two-tailed unpaired *t*-test **(D**, *n* = 11). *P < 0.05, **P < 0.01, and ***P < 0.001. ns, no significance.

To evaluate the expression of IL-21, IL-4, and IFN-γ on CXCR5^+^ or CXCR5^−^CD8^+^ memory T Cells, the mononuclear cells from tonsil tissues, lymph nodes and peripheral blood were isolated and stimulated with PMA plus ionomycin for 6 h in the presence of BFA. FACS results demonstrated that CXCR5^+^ CD8^+^ memory T Cells expressed higher percentages of IL-21, IL-4 and IFN-γ compared with CXCR5^−^CD8^+^ memory T Cells in tonsil tissues and lymph nodes, and the expression of IL-21 and IL-4 in PBMCs was less than that in tonsil tissues and lymph nodes (Figures 3A–D).

**Figure 4:**
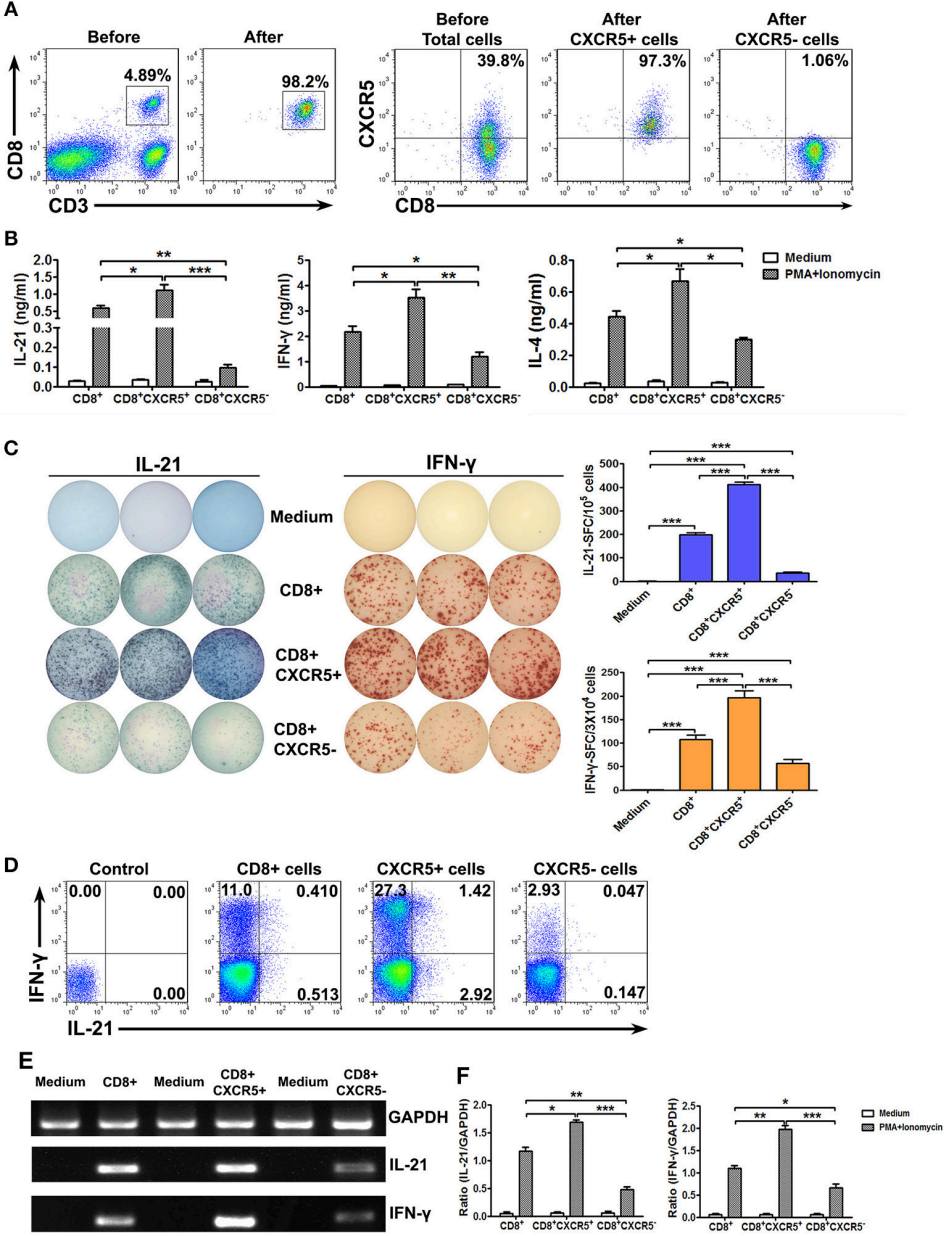
Sorted CXCR5^+^ CD8^+^ T cells expressed higher levels of cytokines at protein and mRNA levels than CXCR5^−^ CD8^+^ T cells. Tonsil CD8^+^ T cells were isolated using the appropriate microbeads, and the purity of the cells was ≥98%. CXCR5^+^ CD8^+^ and CXCR5^−^ CD8^+^ T cells from tonsil CD8^+^ T cells were further sorted by flow cytometry **(A)**. Purified CD8^+^, sorted CXCR5^+^ CD8^+^ and CXCR5^−^ CD8^+^ T cells were stimulated with PMA and ionomycin. The supernatants from the cultures were analyzed by ELISA for the production of IL-21, IFN-γ, and IL-4 **(B)**. The frequency of IL-21 and IFN-γ-producing cells was assessed by ELISPOT. The left panel shows representative counting of spot-forming cells (SFC) and the right panel shows the frequency of IL-21 and IFN-γ-producing cells as the mean with individual data points **(C)**. FACS analysis of IL-21, and IFN-γ expression in CD8^+^, CXCR5^+^CD8^+^, CXCR5^−^CD8^+^ T cells **(D)**. The levels of IL-21, IFN-γ and GAPDH mRNA in CD8^+^, CXCR5^+^CD8^+^, CXCR5^−^CD8^+^ T cells were determined by PCR **(E)**, and the ratio of IL-21 or IFN-γ to GAPDH were quantified by densitometry **(F)**. Statistical significance was compared with Mann–Whitney test. *P < 0.05; **P < 0.01; ***P < 0.001.

To further confirm the ability to produce the cytokines of CXCR5^+^ or CXCR5^−^ CD8^+^ T Cells, we sorted CD8^+^, CXCR5^+^CD8^+^ and CXCR5^−^CD8^+^ T Cells from tonsil tissues by magnetic bead-based and flow cytometric sorting. The purity of CD8^+^, CXCR5^+^CD8^+^, or CXCR5^−^CD8^+^ T Cells was more than 97% and exclude CD4^+^ T Cells (Figure 4A). The cells were stimulated with PMA plus ionomycin for 24 h, the culture supernatants were harvested and detected for the production of IL-21, IL-4, and IFN-γ by ELISA (Figure 4B). Consistent with the results of total cells, CXCR5^+^ CD8^+^ T Cells had more advantages in the production of IL-21, IL-4, and IFN-γ. By using ELISPOT, we also counted a significant number of IL-21 and IFN-γ-producing cells in CXCR5^+^ CD8^+^ T Cell cultures but not in CXCR5^−^CD8^+^ T Cells cultures (Figure 4C). FACS analysis of IL-21 and IFN-γ expression on CD8^+^, CXCR5^+^CD8^+^, or CXCR5^−^CD8^+^ T Cells after stimulation with PMA plus ionomycin showed that the results were as the same as above (Figure 4D). We also demonstrated that CXCR5^+^CD8^+^ T Cells had higher expression of mRNA encoding cytokines including IL-21 and IFN-γ (Figures 4E,F). Overall, those data indicated that CXCR5^+^CD8^+^ T Cells but not CXCR5^−^CD8^+^ T Cells produced cytokines.

### The differentiation of CXCR5^+^CD8^+^ T cells was modulated by transcription factor Bcl-6

To investigate the possible mechanism of the response by CXCR5^+^CD8^+^ T Cells, we evaluated the expression of transcription factors, Bcl-6 and T-bet, in tonsil CD8^+^ T Cells. FACS analysis indicated that there was higher level of Bcl-6 in CXCR5^+^CD8^+^ T Cells than CXCR5^−^CD8^+^ T Cells (*P* < 0.01), and the level of T-bet was no significantly changed between two groups (Figures S2A,B). To determine the regulation of transcription factors to the expression of cytokines, CD8^+^ T Cells were gated according to the expression of IL-21 and IFN-γ. The expression of Bcl-6 and T-bet in each subset was analyzed. The results showed that IL-21^+^IFN-γ^+^ cells expressed the highest levels of Bcl-6 and T-bet in four groups, whereas IL-21-IFN-γ- cells were the lowest one (Figure S2C). The expression of Bcl-6 and T-bet were obviously higher in IL-21^+^CD8^+^ T Cells comparing with IL-21^−^CD8^+^ T Cells. Consistently, the MFI of Bcl-6 and T-bet were higher in IL-21^+^CD8^+^ T Cells than IL-21^−^CD8^+^ T Cells (Figure S2D).

### CXCR5^+^CD8^+^ T cells provide help to B cells for the production of immunoglobulines

**Figure 5:**
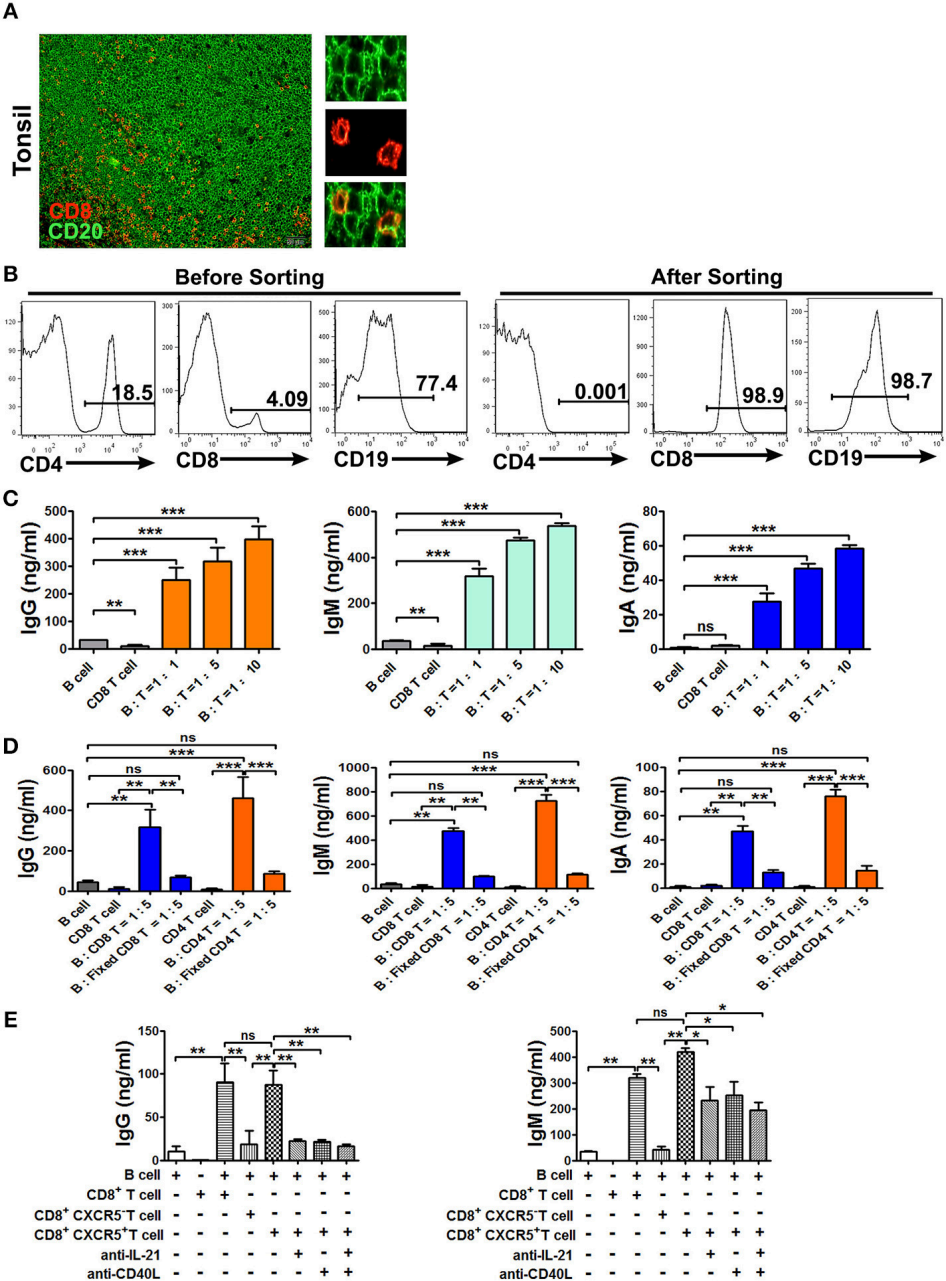
CXCR5^+^ CD8^+^ T Cells provide help to B cells for the production of immunoglobulins. Physical contact between CD8^+^ T Cells (red) and B cells (green) in tonsil sections, and scale bars, 50μm. **(A**, *n* = 5). Sorted tonsil B cells and sorted CD8^+^ T Cells at the ratio of 1:1, 1:5, and 1:10 were co-cultured with or without α-CD3/CD28 dynabeads for 10 days **(B,C)**. Sorted B cells were co-cultured with fresh CD8^+^ or fixed CD8^+^ T Cells, fresh CD4^+^ or fixed CD4^+^ T Cells at the ratio of 5:1 in the presence of α-CD3/CD28 dynabeads for 10 days **(D)**. Sorted B cells and CD8^+^, CXCR5^+^CD8^+^, CXCR5^−^CD8^+^T Cells at the ratio of 5:1 were co-cultured with or without anti-IL-21 and anti-CD40L in the presence of α-CD3/CD28 dynabeads for 10 days **(E)**. The supernatants from the different co-cultures were analyzed by ELISA for the production of IgG, IgM, and IgA. Data are expressed as the mean ± SD, and compared with Mann–Whitney test. *P < 0.05; **P < 0.01; ***P < 0.001; ns, no significance.

The colocalization of B cells and CD8^+^ T cells in tonsil tissues (Figure 5A) prompted us to analyze the effects of CXCR5^+^CD8^+^ T cells on B cells. To this end, we sorted CD8^+^ T cells, CXCR5^+^ and CXCR5^−^CD8^+^ T cells, CD4^+^ T cells and B cells from tonsils by MACS microbeads and FACS (Figure 5B). The cells were cultured over a period of 10 days either B cells alone or B cells with different ratio of CD8^+^ T cells in the presence of α-CD3/CD28 dynabeads. The levels of IgG, IgM, and IgA in co-culture supernatants were assessed by ELISA (Figure 5C). The results showed that a significant increase in IgG, IgM, and IgA production was observed in co-cultures. By contrast, purified B cells co-cultured with fixed CD8^+^ or fixed CD4^+^ T cells at the same condition, and the concentration of IgG, IgM, and IgA in the supernatants were clearly decreased (Figure 5D). To further find out which subsets and molecules of CD8^+^ T cells provided help to B cells, therefore, B cells were co-cultured with CXCR5^+^ or CXCR5^−^ CD8^+^ T cells in the presence or absence of neutralizing Abs to block the effects of IL-21 or CD40L in the culture system. The results showed that a remarkable increase in the IgG and IgM production from B cell and CXCR5^+^CD8^+^ T cell co-cultures compared with CXCR5^−^ T cell. Expression of IgG and IgM were inhibited 40–60% by blocking the production of IL-21 and expression of CD40L (Figure 5E). Taken together, those data demonstrated that CXCR5^+^CD8^+^ T cells in the B cell follicle appear to have an unobtrusive capacity to support B cells through cognate cell-cell contact and production of cytokines.

### IL-21-producing CXCR5^+^CD8^+^ T cells did not express the cytotoxic effector molecules

To characterize cytotoxic factors of CXCR5^+^CD8^+^ T cells, mononuclear cells from tonsils, lymph nodes and PBMCs were stained for the expression of Granzyme B and peforin detected by intracellular stainings. The results showed that CXCR5^+^CD8^+^ T cells expressed higher level of Granzyme B than did CXCR5^−^ CD8^+^ T cells in tonsils and lymph nodes but not in PBMCs. Whereas, CXCR5^+^CD8^+^ T cells were negative for key cytotoxic protein perforin in tonsil and lymph node compared with PBMC (Figures S3A,B). In addition, IL-21-producing CXCR5^+^CD8^+^ T cells did not express the cytotoxic effector molecules (Figure S3C).

### The numbers of CD8^+^CXCR5^+^ cells in the lymph nodes from HIV patients were significantly higher than those in normal individuals

**Figure 6:**
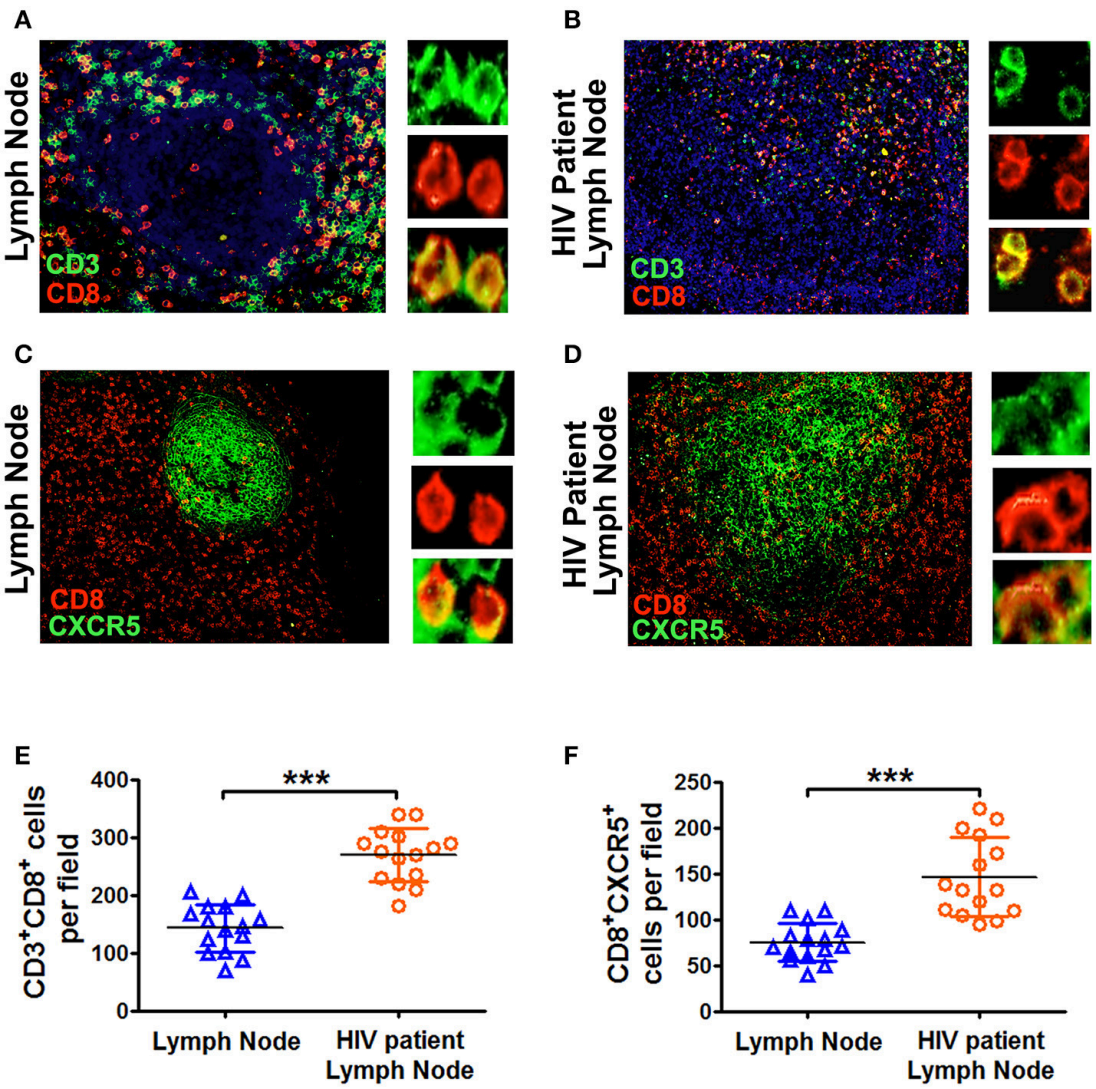
The lymph nodes from HIV patients had more CD8^+^CXCR5^+^ T cells than those from normal lymph nodes. Immunofluorescence of CD3^+^ T cells (green), CD8^+^ T cells (red) and CXCR5 (green) in the germinal centers of lymph node sections (scale bars, 50 μm) from normal individuals **(A,C)** and HIV patients **(B,D)**. The summary data represented the distribution of CD3^+^CD8^+^ T cells and CD8^+^CXCR5^+^ T cells from normal and HIV patient's lymph nodes **(E,F**; *n* = 15), and were analyzed by Mann-Whitney test. ***P < 0.001.

During HIV infection, CD4^+^ cells were gradually diminished in secondary lymphoid tissues. We therefore investigated the distribution of CD8^+^ cells during HIV infection in the lymph nodes. The results showed that there were few CD8^+^ cells in the B cell follicles of normal lymph nodes from HIV^−^ people, but the numbers of such cells were much higher in HIV-infected patients (Figures 6A,B,E). In addition, the numbers of CD8^+^CXCR5^+^ cells was also significantly higher in HIV-infected people than those from normal lymph nodes (Figures 6C,D,F). Those results suggested that in the deficiency of CD4^+^ T cells, CD8^+^ T cells might provide more help to B cells for the production of immunoglobulins.

## Discussion

Classically, CD8^+^ T cells had been found to be largely excluded from lymphoid follicles, being restricted preferentially to extra-follicular areas, providing an ideal environment for immune evasion of a number of pathogens and the generation of oncogenic processes (17, 18, 27–29). However, consistently with the study by Quigley et al. (12), we confirmed that a subpopulation of CD8^+^ T cells, which specifically expressed CXCR5, can migrate to human tonsil and lymph nodes follicle and germinal centers, having an importance role in the maintenance of their architecture, pointing their potential participation in supporting B cells while exerting their classical cytotoxic functions.

The CXCR5^+^CD8^+^ T cells reached the 20~60% of CD8^+^ T cells from tonsils and lymph nodes; by contrast, CXCR5^+^CD8^+^ T cells in peripheral blood were much more sparse than in tonsils, and had a different phenotype in comparison with those confined in lymphoid tissues. Tonsil CXCR5^+^CD8^+^ T cells had high expression of CD45RO and CD27, low level of CD62L and CCR7, suggesting that these cells were antigen-experienced T cells (14, 30). However, the expression of CD62L was high on circulating CXCR5^+^CD8^+^ T cells indicating a capacity to adhere to HEV. One may speculate that the circulating CXCR5^+^CD8^+^ T cells could downregulate follicle homing marker, egress from the follicle, enter to circulation, and possibly migrating to inflamed tissues (15). In addition, there were few CD8^+^ T cells in the B cell follicles of normal lymph nodes, but the numbers of such cells were much greater in HIV-infected people. In contrast, the numbers of CD8^+^ T cells in the T cell zone were similar in normal and patients.

Our results indicated that tonsil CXCR5^+^CD8^+^ T cells were not consistent with a classical cytotoxic cells, and IL-21-expressing tonsil CXCR5^+^CD8^+^ T cells did not express granzyme B and perforin. Tonsil CXCR5^+^CD8^+^ T cells and circulating CXCR5^+^CD8^+^ T cells both were almost negative for the key cytolytic protein perforin, but the expression of granzyme B was higher in both population. It is possible that CXCR5^+^CD8^+^ T cells possess cytotoxic effector but a subset of CXCR5^+^CD8^+^ T cells in germinal centers of lymph nodes constitute non-fully differentiated follicular CD8^+^ T cells that have not reach the cytotoxic phenotypic and functions (19). During polyclonal activation, CXCR5^+^CD8^+^ memory T cells produced more IL-21, IL-4 and IFN-γ compared with CXCR5^−^CD8^+^ memory T cells in tonsils and lymph nodes. In contrast, the expression of IL-21 and IL-4 in periphery CD8^+^ T cells were much more scarce than that in lymphoid tissues. Furthermore, an increase in the production of antibodies by B cells when B cells were co-cultured with CD8^+^ T cells or CXCR5^+^CD8^+^ T cells, was not observed with fixed CD8^+^ T cells or CXCR5^−^CD8^+^ T cells. At least in part, this effect is due to the production of IL-21 by CXCR5^+^CD8^+^ T cells, a cytokine typically produced by Tfh cells which, among other functions, promotes the differentiation of B cells and antibody production (31–34). This speculation was confirmed when the production of IgG and IgM was inhibited by blocking the production of IL-21. Notably, the expression of CD40L and ICOS by CXCR5^+^CD8^+^ T cells was higher compared with CXCR5^−^CD8^+^ T cells, suggesting that a receptor-mediated cooperative interaction between the CXCR5^+^CD8^+^ T cells and B cells (35–38). Together, these data indicated that CXCR5^+^CD8^+^ T cells might resemble Tfh cells in the ability to support B cells and promote antibody responses.

Since the last decades, numerous studies have shown that Bcl-6 is the classical transcription factor of Tfh cells and critical for the formation of B cells in the germinal centers (38–41). Our results also showed that higher expression of Bcl-6 in CXCR5^+^CD8^+^ T cells and IL-21-producing CD8^+^ T cells. In addition, Bcl-6 is upregulated in memory CD8^+^ T cells and suppresses granzyme B expression (42). In line with that notion, our data suggested that IL-21-producing CXCR5^+^CD8^+^ T cells did not co-express the cytotoxic molecules. Thus, due to the expression of Bcl-6, CXCR5^+^CD8^+^ T cells possess follicular helper-like characteristics, but potentially decreased cytotoxic functions.

It has to be noted that we only use human subjects to describe the phenotype and functional profiles of CXCR5^+^CD8^+^ T cells. Future research should include animal models with the depletion of total CD8^+^ T cells to discuss the effect of CD8^+^ T cells on the generation of germinal centers and antibody production by B cells.

In summary, we have currently confirmed a subset of human CD8^+^ T cells in tonsils and lymph nodes expressed CXCR5 to localize in B cell follicles. CXCR5^+^CD8^+^ T cells displayed effector or central-memory phenotype, expressed CD40L, produced IL-21 and might be similar to Tfh cells in the ability to provide help to stimulate B cells and to promote antibody responses.

## Author contributions

JS performed most experiments and analyzed data with the support from CW; XL, and QW performed flow cytometry on tonsil samples; JH and GX performed immunofluorescence staining and quantification of HIV samples; LW, HL, and BY contributed to scientific planning; and CW oversaw and designed the study. JS and CW wrote the manuscript.

### Conflict of interest statement

The authors declare that the research was conducted in the absence of any commercial or financial relationships that could be construed as a potential conflict of interest.

## Supplementary material

Figure S1 CXCR5^+^ CD8^+^ T cells expressed effector- and central-memory phenotypes. Tonsil cells without stimulation were stained and analyzed by flow cytometry for expression of memory makers. The representative graphs are gated on CD3^+^ CD8^+^ T cells, CXCR5^+^ CD8^+^ T cells and CXCR5^−^ CD8^+^ T cells **(A)**. The representative dot plots show the expression of CD45RO, CCR7, CD62L and CD27 in CXCR5^+^ or CXCR5^−^ CD8^+^T cells **(A–C)**. Data represent mean ±SD of five separate experiments, and compared with two-tailed unpaired *t*-test **(C**, *n* = 5). ^*^*P* < 0.05 and ^**^*P* < 0.01, and ns, no significance.

Figure S2 The expression of transcriptional factors in CXCR5^+^ and CXCR5^−^ CD8^+^ T cells. Tonsil cells were stimulated with PMA and ionomycin in the presence of BFA for 6 h. The expression of Bcl-6 and T-bet from CXCR5^+^ and CXCR5^−^CD8^+^ T cells were assessed by intracellular staining. The representative histogram graph and summary data were shown **(A,B**, *n* = 4]. CD8^+^ T cells were gated according to the expression of IL-21 and IFN-γ in tonsils. The expression of Bcl-6 and T-bet in each subset was analyzed **(C,D)**. Data were representative of five separate experiments, and compared with two-tailed unpaired *t*-test. ^**^*P* < 0.01 and ^***^*P* < 0.001. ns, no significance.

Figure S3 The expression of cytolytic molecules by CXCR5^+^ CD8^+^ T cells from tonsils, lymph nodes and PBMCs. Mononuclear cells from tonsils, lymph nodes and PBMCs without stimulation were analyzed for the expression of granzyme B and perforin by flow cytometry **(A)**. The representative histogram graphs and summary data were shown (**B**, *n* = 5). Tonsil cells were stimulated with PMA and ionomycin in the presence of BFA for 6 h. The expression of IL-21 and granzyme B was analyzed by FACS **(C)**. Data are expressed as the mean ± SD, and compared with Mann-Whitney test. ^*^*P* < 0.05; ^**^*P* < 0.01; ns, no significance.
