# Supplementary figures and images for: A Subset of CXCR5+CD8+ T Cells in the Germinal Centers From Human Tonsils and Lymph Nodes Help B Cells Produce Immunoglobulins
Source: Front Immunol. 2018 Oct 5;9:2287. doi: 10.3389/fimmu.2018.02287 (PMC6183281; doi:10.3389/fimmu.2018.02287)

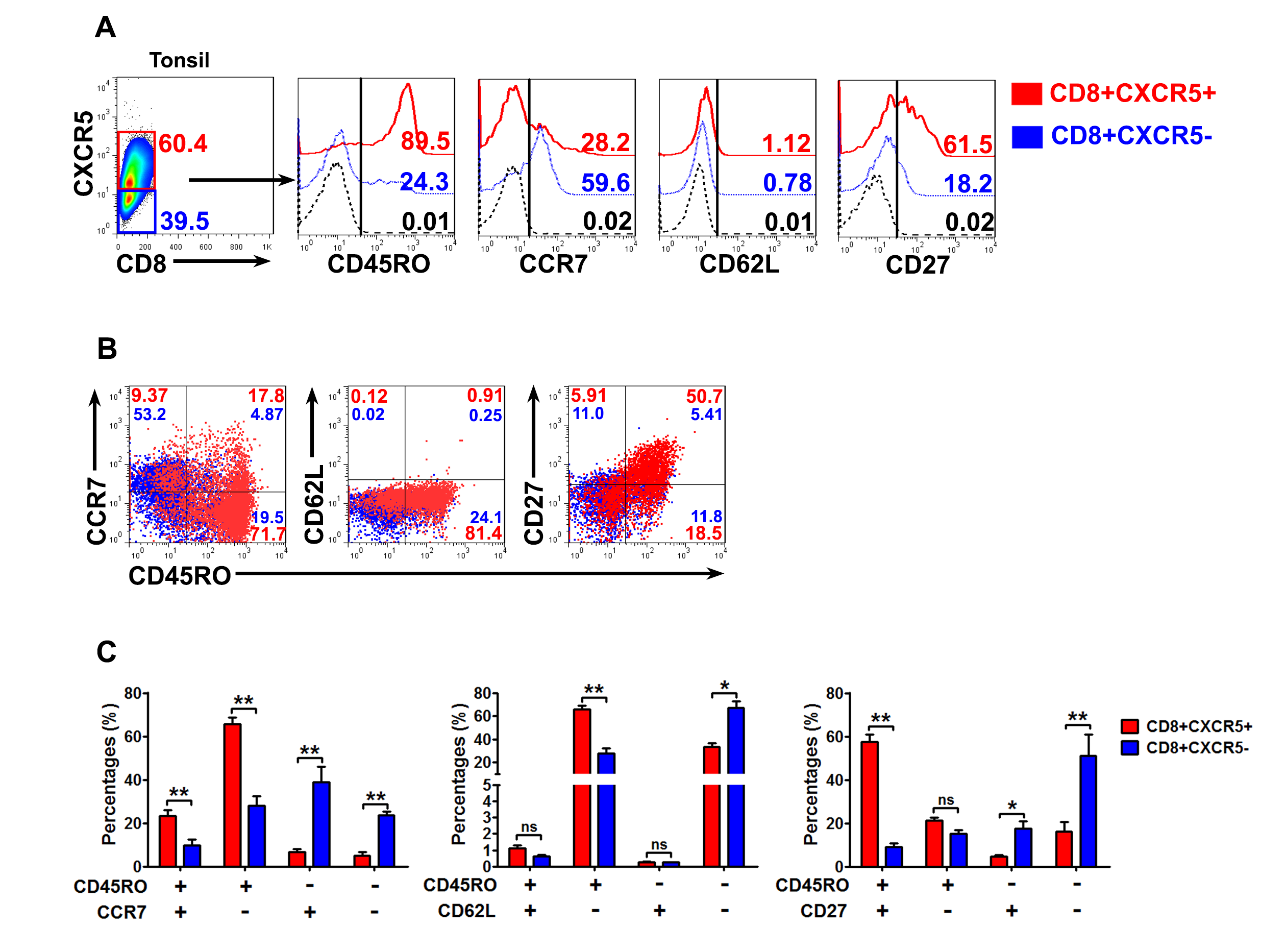

Supplement: Figure S1 — CXCR5+ CD8+ T cells expressed effector- and central-memory phenotypes. Tonsil cells without stimulation were stained and analyzed by flow cytometry for expression of memory makers. The representative graphs are gated on CD3+ CD8+ T cells, CXCR5+ CD8+ T cells and CXCR5− CD8+ T cells (A). The representative dot plots show the expression of CD45RO, CCR7, CD62L and CD27 in CXCR5+ or CXCR5− CD8+T cells (A–C). Data represent mean ±SD of five separate experiments, and compared with two-tailed unpaired t-test (C, n = 5). *P < 0.05 and **P < 0.01, and ns, no significance. [file Image_1.TIF]

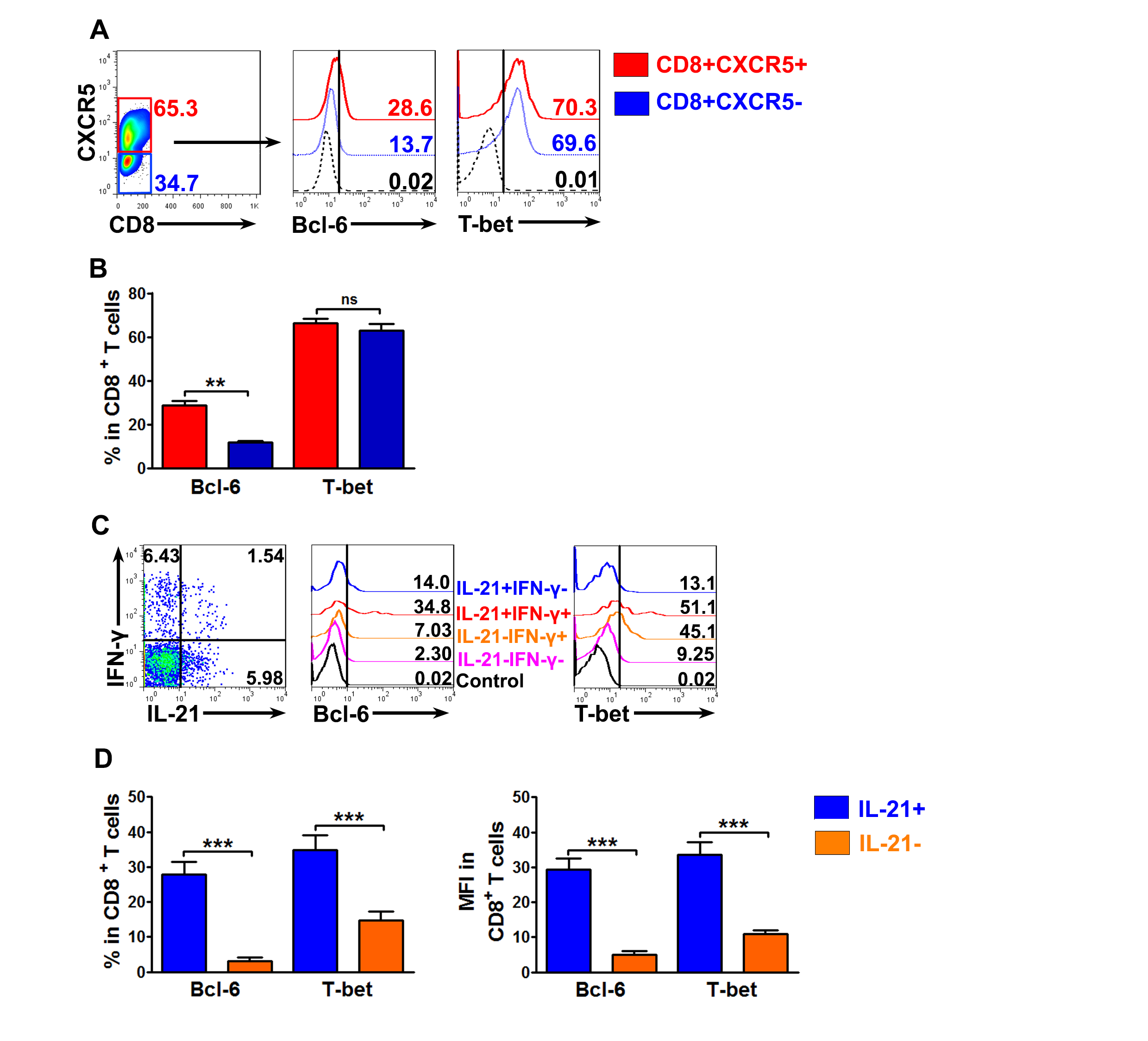

Supplement: Figure S2 — The expression of transcriptional factors in CXCR5+ and CXCR5− CD8+ T cells. Tonsil cells were stimulated with PMA and ionomycin in the presence of BFA for 6 h. The expression of Bcl-6 and T-bet from CXCR5+ and CXCR5−CD8+ T cells were assessed by intracellular staining. The representative histogram graph and summary data were shown (A,B, n = 4]. CD8+ T cells were gated according to the expression of IL-21 and IFN-γ in tonsils. The expression of Bcl-6 and T-bet in each subset was analyzed (C,D). Data were representative of five separate experiments, and compared with two-tailed unpaired t-test. **P < 0.01 and ***P < 0.001. ns, no significance. [file Image_2.TIF]

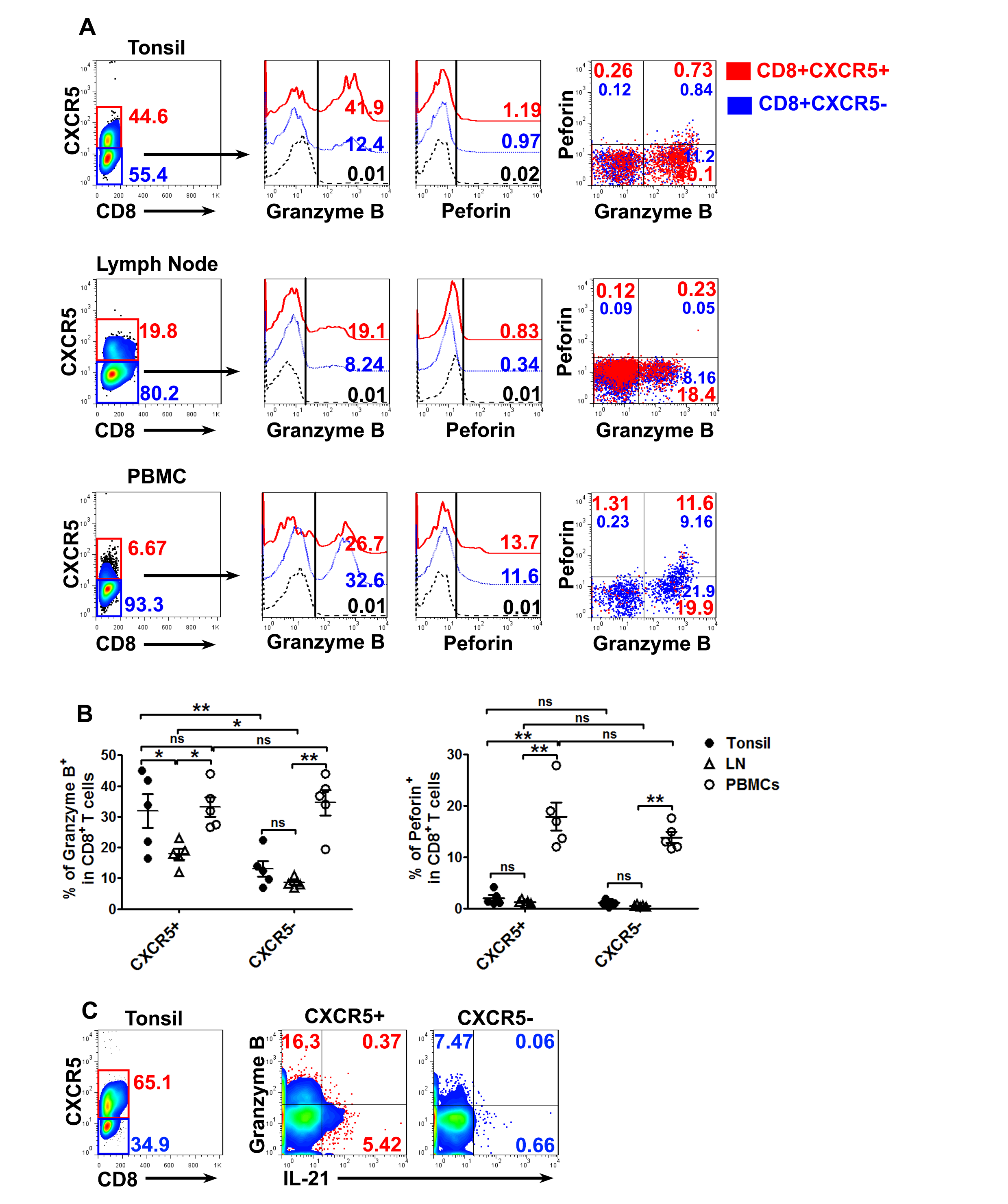

Supplement: Figure S3 — The expression of cytolytic molecules by CXCR5+ CD8+ T cells from tonsils, lymph nodes and PBMCs. Mononuclear cells from tonsils, lymph nodes and PBMCs without stimulation were analyzed for the expression of granzyme B and perforin by flow cytometry (A). The representative histogram graphs and summary data were shown (B, n = 5). Tonsil cells were stimulated with PMA and ionomycin in the presence of BFA for 6 h. The expression of IL-21 and granzyme B was analyzed by FACS (C). Data are expressed as the mean ± SD, and compared with Mann-Whitney test. *P < 0.05; **P < 0.01; ns, no significance. [file Image_3.TIF]
